# Pneumonia

**Published:** 1855-10

**Authors:** 


Pneumonia.—Prof. Flint made to the Buffalo Medical Association, in September, an elaborate and highly interesting report on the pathology, prognosis and treatment of Pneumonia, from which we make the following extract:

In regard to the effect of blood-letting on the duration of pneumonia, the papers referred to the committee, communicated to the association by a member of the committee, contain certain facts which are to be noticed. Four cases of pneumonia are reported in which blood-letting was employed in all on the fourth day; in two of the cases being practiced three times, and in two once only. In one of the cases the patient was a child 4½ years of age. The duration of the disease in these cases, respectively, are are given as follows: 8, 5, 4, 2½ days. The mean duration is about 8¾ days. This is a small fraction below the mean duration in Dr. Ames's fifty-one cases treated with neither bleeding, antimony, nor mercury. The cases reported by a member of this committee, Dr. Burwell, were not given as illustrating a mode of practice to be pursued indiscriminately, or even generally, in the treatment of pneumonia; but as evidencing the success following blood-letting under circumstances which appeared to him to indicate in a special manner the employment of this measure. I shall not discuss the propriety of the practice pursued in these cases. Considering their terminatioon and duration, the only question for discussion is, whether the detraction of blood was necessary; in other words, whether the favorable progress of the disease was not due to its natural tendencies irrespective of the treatment. This is a question more easily discussed than answered.

In connection, however, with the results in the cases reported by my associate on the committee, I will give the duration in six cases occurring during the present summer, and treated by me, with the exception of one case which I saw in consultation with Dr. Samo, but observed, and of which I took notes during the whole career of the disease. Five of these cases were male patients at the Hospital of the Sisters of Charity, and in the remaining single case the patient was a child, five years of age, treated in private practice. These are all the cases which I have observed during the present summer, in which blood-letting had not been practiced prior to their coming under my observation. In the case of the child a little antimony was prescribed. In this case the pneumonia was lobar, and uncomplicated with bronchitis. In all the other cases the treatment consisted of anodynes and stimulants. All the cases are fully recorded. In three of the cases an entire lung was consolidated. In the three remaining cases the inflammation was limited to the lower lobe. The cases came under observation at periods from the date of the attack as follows: 5, 3, 8, 8, 6, 1 days. Reckoning from the initial chill, to a period when the pulse had fallen to the natural standard, or below it, which not infrequently occurs, and the respirations but little, if at all accelerated, the duration in the cases respectively, was as follows: 11, 7, 14, 12, 7, 5. The mean duration is precisely 9⅓ days. The average duration exceeds that in the cases reported by Dr. Burwell, by a fraction less than a day. It is to be borne in mind that my cases were, with one exception, hospital cases; that the patients entered at a period from the date of attack, varying from three to eight days, had received no treatment prior to their admission, and in one-half the numbhr of cases the inflammation extended over the entire lung on one side. In each of the cases the recovery was rapid. The duration to the time of being dressed, in the cases severally, is as follows: 14, 9, 20, 21, 8, 6 days. In three cases the patients remained in hospital for some time after being able to be discharged, two of them being employed as out-door assistants. The remaining two patients were discharged each on the fifteenth day from the date of the attack. Medical attendance on the child was discontinued on the eighth day.

The few cases reported by Dr. Burwell and myself, thus present a mean duration not far from equal; the former treated with and the latter without bleeding. In neither collection is the number of cases sufficiently large to warrant statistical deductions; but they serve, exhibited in contrast, to exemplify the general conclusion at which we must arrive after a consideration, be it ever so elaborate, of the evidence for or against the employment of blood-letting in pneumonia, namely, the propriety of this measure of therapeutics must be determined by the practitioner in each case separately, not by following any fixed formula; but by a rational interpretation of the indications which cases individually offer, guided by certain general principles regulating the employment of blood-letting in all acute inflammations. This conclusion embraces postulates, which to unfold and treat of folly would require more space than falls within the proper limits of this report. Assuming that we should be led to it had we time to follow the chain of considerations by which it is reached, diversity of practice among practitioners hinges mainly on the different views entertained respecting the general principles regulating the employment of blood-letting in all acute inflammations. Here, again, is a broad field of inquiry involved in a full investigation of the present subject. It will not, of course, be expected that I should enter upon it; but it *will* probably be expected that I shall not leave the subject without farther expression of my views respecting the treatment of pneumonia by blood-letting. My remarks already occupy so much space that I ought not to presume on your attention by extending much farther this report; and I shall, in conclusion, for the sake of brevity, embrace in a series of propositions certain practical points of consideration, which, if my leisure and your interest permitted, I should present in a shape not to be disposed of so cursorily. As it is I can only add a very few brief comments, leaving a fuller exposition for oral discussion.

1. Primary pneumonia in the form in which it is usually presented in the adult, namely, confined to the lower lobe of one lung, tends so uniformly to recovery, that interference by blood-letting is not requisite as a means of securing a favorable issue of the disease. The objects, then, for which blood-letting is practiced in such cases must be embraced in the following enumeration: *a.* To arrest the disease. *b.* To abridge its duration. *c.* To limit the diffusion of the inflammation. *d.* To lessen the local morbid effects incident to pulmonary inflammation. *e.* To relieve pain and other distressing symptoms. *f.* To promote rapid convalescence and the complete restoration to health.

2. It appears to be fully established that blood-letting does not possess the power of arresting the disease. Its employment with a view to this object is therefore unwarrantable, and, if not otherwise indicated, must do harm in proportion to the want of ability on the part of the patient to bear up under the effects of so potent a therapeutical measure.

3. Clinical facts appear to show that in a series of cases bloodletting exerts a limited influence in shortening the duration of pneumonia. We have not, however, evidence that this influence is exerted save in a ceriain proportion of cases, and it by no means authorizes the employment of blood-letting in cases indiscriminately. The effect on the duration of the disease is to be regarded as incidental to its judicious employment under other indications. To bleed every patient because it has been found that in a large collection of cases the average duration is less in the cases bled than in those not bled, is a vicious application of statistical results. To abridge the duration of the disease, is, therefore, not a legitimate object of taking blood, disconnected from other objects.

4. The extension of inflammation from one lobe to another in the same side, or in the opposite side, which occurs in a certain proportion of cases, adds to the gravity of the disease, and it is, of course, desirable to prevent its occurrence. We have not, however, positive evidence that blood-letting possesses any power to limit the diffusion of the inflammation. There is reason to believe that when the inflammation subsequently invades other lobes than the one primarily attacked, it is due to an internal tendency which, with our present knowledge and resources, we are unable to control. It is probable that instances in which an entire lung is affected, or in which the pneumonia is double, would be found not less numerous among a certain number of patients bled, than among the same number not bled. This is an interesting point to be settled by statistical researches.*

5. The local morbid changes incident to inflammation, namely, inflammatory exudation leading to obstruction of the air resides and interruption of the circulation through the parts inflamed, occur very uniformly in pneumonia, and, generally, early in the disease. It must be considered doubtful whether, to much extent, these changes may be lessened by blood-letting. The problem is a difficult one to determine, on the one hand, in cases in which blood-letting is practiced, whether the local changes are less than they would have been had no blood been taken ; and, on the other hand, in cases in which blood-letting is not practiced, whether the changes are greater than they would have been had this measure been employed. Considering that the physical signs of solidification are generally present in cases bled, as well as in those in which blood-letting is not practiced, the effect of the measure in limiting the amount of exudation is probably small, and exerted in only a certain proportion of cases. While we are not warranted in denying that blood-letting ever lessens the local changes in pneumonia, the grounds for expecting this result are not suffi-

* Whether this point has been made the subject of statistical investigation, or whether data for such an investigation are available, the writer, at the moment of preparing this report, is unable to state.

cient to constitute it an object for taking blood under circumstances when, if this effect be not produced, the condition of the patient will be damaged. It is obvious that with reference to this object any efficiency which blood-letting possesses depends on its employment within the first two, three or four days.

6. To relieve pain and other distressing symptoms is a legitimate object of therapeutics, and that blood-letting in certain cases is followed by a marked abatement of the local symptoms cannot be doubted. In resorting to paliative measures, however, their ulterior effects are not to be overlooked. It would manifestly be bad practice to incur a risk of doing harm remotely, in order to secure a temporary alleviation of pain or distressing symptoms. Blood-letting, then, may be practiced early in pneumonia with a view to relieve the acute pain, and labored respiration present in some cases, but not under cicumstances contra-indicating it, and not carried to an extent which will be likely to involve ulterior damage.

7. The objects to be attained by blood-letting, it is evident, relate to the existing disease. No one, it is to be presumed, advocates taking blood without reference to present indications or the immediate effects, but with a view to its influence on convalescence and the completeness of the recovery. On the other hand, the influence which the loss of blood may have in retarding convalescence, and leaving the system more enfeebled than it would have been from the effects of the disease alone, should restrain the practitioner from employing blood-letting in some instances, when, without any regard to its remote consequences, it might be indicated. The evils of unnecessary and excessive blood-letting in the disease are probably manifested by slow and incomplete restoration to health, rather than by an increased rate of mortality.

8. In view of the considerations contained in the preceding propositions, the objects for which blood-letting is practiced in pneumonia are few and limited; and in resorting to it the practitioner must be guided by circumstances pertaining to cases individually, and by the general principles regulating its application to acute inflammation. These circumstances and principles will supply the subjects of the few remaining propositions.

9. The circumstances indicating blood-letting in pneumonia, as the rule, belong only to an early period of the disease. Indications are rarely presented after solidfication has occurred. The processes of restoration depending on the energies of the system are not likely to be favorably affected by the loss of blood; but, on the contrary, the recuperative power may be diminished.

10. General indications for blood-letting in acute inflammations have relation to circumstances incidental to the inflammation rather than to the inflammation itself. They relate to the degree and character of the febrile movement; to the previous condition of the system; to the habits and constitution of the patient. Special indications in pneumonia relate to pain and embarrassment of respiration.

11. In pneumonia, as in acute inflammations generally, blood-letting is indicated, as the rule, only when symptomatic fever is present, and of a high grade, as denoted by increased frequency, and force of the pulse, the symptoms showing not simply morbid action, but augmented power of the circulation.

12. Polyæmia or plethora, previous good health, and vigor of constitution, are circumstances rendering blood-letting safe and proper; when, *per contra*, anæmia, feebleness of constitution, or impaired health from former disease, deprivations, over exertion, intemperance, etc., etc., in connection with the same degree and extent of local inflammation, render it inappropriate and perhaps unsafe.

13. In conclusion, blood-letting in pneumonia, as in other acute inflammations, in some instances not only conduces to immediate relief, but abates by an indirect action the intensity of the inflammation, and exerts a favorable influence on the progress of the disease. But in this, as in other affections, indiscriminately employed, or carried to an injudicious extent, it is a measure potent for harm, and may prove destructive. Employed in a manner to secure its benefits and avoid its evils, it is a useful remedy in a certain proportion of cases; and this proportion would be found to be not uniform in patients of different conditions; in the residents of a city and the country; in different countries and in different portions of the same country, and in successive years. Of cases occurring at the same time and in the same locality, in some it will be useful, in others hurtful. A just appreciation of the principles and rules which should regulate its employment, is essential; but in their application to individual cases, the line of practical conduct cannot be formularized, but must be left to the judgment, and perhaps it may be said the sagacity, which, in addition to scientific attainment, are necessary to form the skillful physician.

It will be observed that the remarks contained in the foregoing propositions have reference to primary, uncomplicated pneumonia in the adult. The applicability of blood-letting, as a general remark, is less when the disease is secondary, or complicated, or occurring at either of the extremes of life.—*Buffalo Med. Jour.*

